# Effect of Combining Germination and Heat–Moisture Treatment of Unpolished Red Rice on the Physicochemical Properties and Digestibility of Its Starch

**DOI:** 10.1155/ijfo/6019328

**Published:** 2025-10-24

**Authors:** Tran Ngoc My Linh, Le Thi Thu Suong, Nguyen Thi Lan Phi, Nguyen Ngoc Thanh Tien, Mai Nguyen Tram Anh, Chau Minh Thuan, Pham Van Hung

**Affiliations:** ^1^ Department of Food Technology, International University, VNU-HCM, Linh Xuan Ward, Ho Chi Minh City, Vietnam, hcmiu.edu.vn; ^2^ Vietnam National University, Ho Chi Minh City, Vietnam, vnu.edu.vn; ^3^ Faculty of Chemical Engineering, Ho Chi Minh City University of Technology (HCMUT), Ho Chi Minh City, Vietnam, hcmut.edu.vn; ^4^ Laboratory for Interdisciplinary Research and Analysis on Chemistry, Food and Environment (LIRACFE), International University, VNU-HCM, Ho Chi Minh City, Vietnam, hcmiu.edu.vn

**Keywords:** germination and heat–moisture treatment, physicochemical properties, red rice, starch crystallinity, starch digestibility

## Abstract

This study investigated changes in the physicochemical characteristics of starches obtained from unpolished red rice (URR) under germination and combined germination and heat–moisture treatment (HMT). Starches isolated from native, germinated, and germinated combined with HMT exhibited high purity, an A‐type crystalline structure, and irregular polyhedral granules measuring 3–8 *μ*m in size. Germination did not influence the pasting temperature, trough and final viscosities, or setback of red rice starch. However, it increased the maximum viscosity, breakdown, and swelling power while reducing solubility. Germination elevated the concentration of rapidly digestible starch (RDS) and decreased the amount of resistant starch (RS). Notably, the increments in RDS and reductions in slowly digestible starch (SDS) and RS were positively correlated with longer germination durations (6–24 h). When HMT was applied after germination, increases were observed in pasting temperature, solubility, and RS content, while decreases occurred in maximum viscosity, breakdown, swelling power, and RDS content. Consequently, starches extracted from red rice germinated for 6 h and subsequently modified by HMT exhibited desirable characteristics, including the lowest RDS content (54.09%) and the highest RS content (30.39%) among all tested samples. These starches could be utilized in the production of low‐carb products.

## 1. Introduction

Unpolished red rice (*Oryza sativa* L.), which retains all its bran layer, embryo, and endosperm, is rich in nutritional and biofunctional components, including protein, vitamins, dietary fibers, minerals, proanthocyanidins, *γ*‐aminobutyric acid (GABA), and *γ*‐oryzanol [1]. Notably, red rice polyphenols exhibit strong antioxidant capacity [2], antitumor effects [3], hypoglycemic effects, and inhibition of pancreatic *α*‐amylase activity [4]. Consumption of red rice offers significant health benefits, including potential protection against celiac disease [5]. Consequently, this grain is consumed directly as cooked whole rice or utilized as a natural ingredient in commercial food products such as crackers, pasta, rice milk, or fermented wine [6]. Recent studies have indicated that germination is an effective, simple, and affordable method for enhancing the flavor, digestibility, and nutrient availability of cereal grains [7]. This biochemical process primarily activates endogenous enzymes, which break down macromolecules into smaller molecular components [8]. Tian et al. [9] reported that germinated rice contained higher levels of fibers, minerals, vitamins, ferulic acid, and *γ*‐oryzanol compared to native rice. Additionally, the accumulation of new bioactive compounds, such as tocopherols, tocotrienols, GABA, and *γ*‐oryzanol, was observed during germination [10, 11]. Ding et al. [1] found that red rice achieved its peak GABA concentration, approximately 750 mg/kg, after 66 h of germination.

Although extensive research has focused on improving the nutritional value and health benefits of red rice through germination, previous studies confirm that starches obtained from germinated rice are more soluble and digestible than those isolated from raw rice [12]. Specifically, starch undergoes partial or complete hydrolysis during germination, leading to structural modifications such as increased erosion of starch granules, decreased relative crystallinity, and a reduced degree of short‐range order [13]. Consequently, germinated rice exhibits a high glycemic index, making it unsuitable for diabetic and overweight individuals. Recently, our published work demonstrated that heat–moisture treatment (HMT) applied directly to unpolished red rice significantly altered the format and degree of agglomeration of starch granules, suppressed swelling and breakdown of starch paste, enhanced resistant starch (RS) content, and reduced postprandial blood glucose levels in mice [14]. Similarly, Chung et al. [15] found that while germination significantly reduced pasting properties and increased starch digestibility in brown rice, HMT of germinated brown rice (GBR) improved pasting viscosity and gelatinization temperatures, reduced rapidly digestible starch (RDS) content, and increased concentrations of slowly digestible starch (SDS) and RS. Thus, the short‐chain starch molecules of germinated rice, produced by the action of amylase on starch granules during germination, are reassociated after HMT to change starch digestibility. However, the impact of a combination of germination and HMT on the production of the short‐chain starch molecules and modification of the structure and physicochemical properties of starch is not found in the literature. Therefore, the objective of this study is to investigate the effects of germination at varying durations (0, 6, 12, 18, and 24 h) and the combined effects of germination and HMT on the structural, physicochemical characteristics, and digestibility of starches isolated from unpolished red rice after germination and HMT.

## 2. Materials and Methods

### 2.1. Materials

*Riz flottant* variety of red rice (*Oryza sativa* L.), whose origin was from Tien Giang Province, Mekong Delta, Vietnam, was a commercial commodity supplied by Tuan Mui Rice, Ho Chi Minh City, Vietnam. The authentication of this material was 3962/PKN‐VKNQG issued by the National Institute for Food Control of Vietnam. All grains chosen for this study should be free of contamination and kept at room temperature until utilization. Alpha‐amylase from *Aspergillus oryzae* (~30 U/mg, Product#10065), amyloglucosidase from *Aspergillus niger* (≥ 300 U/mL, Product#A7095), and all analytical reagents were bought from Sigma‐Aldrich Co. (St. Louis, Missouri, United States).

### 2.2. Germination and HMT of Unpolished Red Rice Grains

The germination procedure was conducted based on the reports of Hung et al. [14] and Yen et al. [16] with minor adjustments. Firstly, the mixture of unpolished red rice and water (1:4, *w*/*v*) was prepared in a 250‐mL glass container and submerged for 1 h, and then the grains were drained off. Next, the samples were immersed in a 0.05% NaOCl solution for 10 min to sterilize the grain surface and prevent microbial development, followed by rinsing multiple times in tap water. Then, the grains were steeped in tap water at the ratio of 1:4 (*w*/*v*) at ambient temperature for 8 h. Afterwards, the hydrated rice grains were germinated in an automatic germination machine (BKCN1, Hanoi, Vietnam) at room temperature for 6, 12, 18, or 24 h. After germinating, the germinated rice grains were dried in a forced draft oven at 50°C until their moisture content reached lower than 12% and then were ground into fine flour before being stored for further utilization. A control sample was also prepared using a similar procedure without germination.

HMT was applied on germinated red rice according to the previous study of Hung et al. [14]. The germinated rice grains were firstly premoisturized to a level of 30% in an airtight Erlenmeyer flask, equilibrated at ambient temperature for 24 h, and then incubated at 100°C for 6 h. After HMT, the grains were dried in a forced air oven at 50°C for 24 h until the moisture was 10%–12% and then ground into fine flour before being stored for further utilization.

### 2.3. Isolation of Starch From Red Rice Grains

Starches isolated from flours of the germinated rice grains or the HMT germinated rice grains were conducted based on the procedure of Hung et al. [14]. The flours (50 g) were firstly mixed with 400 mL of 0.2% NaOH and then incubated at 4°C overnight. The slurry was centrifuged at 3000 × g for 20 min, and then the supernatant was finally drained off to remove protein. This submersed stage was repeated twice, and the sedimentation was washed multiple times with distilled water. Afterwards, the starch slurry was filtered through a 100‐mesh screen and then recovered by centrifugation (4500 × g for 10 min). After drying in a forced air oven at 45°C until the moisture content of final products reached lower than 11%, the dried starch was stored for further measurement.

### 2.4. Proximate Analysis of Isolated Starches

The standard AACCI approved procedures were employed to measure the concentrations of lipid (30‐10), protein (46‐10), and ash (08‐01) of isolated starches [17]. In addition, the total carbohydrate content was estimated by subtracting all protein, lipid, and ash contents from the whole composition (100%).

### 2.5. Morphological and Crystalline Structures of Red Rice Starches

Morphological structure of isolated starches was determined utilizing a scanning electron microscope (Model S‐3700N, Hitachi, Japan). The photograph was taken at an acceleration potential of 20 kV according to the report of Hung et al. [14].

Crystalline structure of isolated starches was investigated by an x‐ray diffractometer (Rigaku Co. Ltd., Rint‐2000 type, Tokyo, Japan) according to the procedure of Hung et al. [14] with minor modifications. The scanning program was operated at the range of 5° 2*θ*–35° 2*θ*, the velocity of 4^o^/min, and the step of 0.02° with the fixed voltage (40 kV) and current (80 mA).

### 2.6. Pasting Properties, Swelling Power, and Solubility of Isolated Starches

A micro‐viscoamylograph (Brabender GmbH & Co. KG, Germany) was applied to determine the pasting properties of isolated starches according to the procedure of Hung et al. [14]. The mixture of starch and water (8%, *w*/*v*) was firstly prepared, raised temperature from 30°C to 93°C at a heating speed of 7.5°C/min, remained at 93°C for 15 min, then cooled to 30°C at the same rate, and finally endured at 30°C for 15 min. The viscoamylograph of starch pastes was automatically recorded and expressed as pasting temperature, maximum viscosity, trough viscosity, final viscosity, breakdown, and setback.

Swelling power and solubility of red rice starch were evaluated according to the procedure revealed by Trung et al. [18]. Regarding the determination of the swelling power, 0.16 g of starch was first mixed with 5 mL of water and then incubated at defined temperatures (50, 60, 70, 80, and 90°C) for 30 min before cooling down, centrifuging at 3000 × g for 15 min and collecting the residue. The swelling power was computed by dividing the mass of the residue by the mass of the sample. Regarding the determination of the solubility, a starch suspension (0.50 g of starch in 30 mL of water) was boiled at certain levels (50, 60, 70, 80, and 90°C) for 30 min before cooling down, centrifuging at 1500 × g for 30 min and collecting the supernatant, which was then evaporated at 120°C for 4 h. The solubility was estimated by calculating the ratio of the mass of starch in the supernatant to the mass of the original sample.

### 2.7. In Vitro Digestibility of Red Rice Starch

In vitro digestibility of isolated starches, expressed as RDS, SDS, and RS, was determined based on the method of Englyst et al. [19] with minor modification. A starch suspension (0.30 g of starch in 20 mL of sodium acetate buffer) was boiled for 30 min, stabilized at 37°C for 15 min, mixed with a 5 mL solution of *α*‐amylase (1400 U/mL) and amyloglucosidase (13 AGU/mL), and finally incubated at 37°C. Afterwards, the analyzed solution was sequentially withdrawn after hydrolyzing for 20 and 120 min and was then treated with the phenol–sulfuric acid method to determine the total amount of glucose that existed in the hydrolysate (G20 and G120, respectively). The remaining liquid after incubating for 120 min was used for the measurement of total glucose concentration after mixing with 7M KOH, followed by hydrolyzing with amyloglucosidase (50 AGU/mL). Consequently, G20, G120, and TG were employed to estimate RDS, SDS, and RS.

### 2.8. Statistical Analysis

Data was recorded from at least triplicates and was revealed as the median values. The statistical analysis was handled through analysis of variance and Tukey′s post hoc test with *p* < 0.05 by SPSS software (IBM SPSS Statistics 22, New York, United States).

## 3. Results and Discussion

### 3.1. Proximate Analysis of Rice Starches

Table 1 shows the proximate composition of starches isolated from unpolished red rice after combining germination and HMT. All isolated starches contained low amounts of protein (0.02%–1.81%, db), lipid (0.08%–0.85%, db), and ash (0.33%–1.56%, db), indicating that the purity of the obtained product was high, with total carbohydrate concentrations ranging from 96.8% to 99.4% (db). These findings were consistent with previous literature, which revealed that rice starch contains 0.07%–0.68% protein, 0.01%–0.35% ash, and approximately 98% starch [20]. Wang et al. [13] also reported that germinated red rice starches contained less than 0.5% protein and a low percentage of lipid and ash, suggesting that these isolated starches could serve as a source of high‐purity starch. In this study, the high protein content (1.815%, db) of the starch isolated from the 24‐h‐germinated rice following HMT (GH‐RS‐24h) might be attributed to protein denaturation and interactions between protein and starch chains, forming starch–protein complexes after germination and HMT [21].

**Table 1:** Proximate analysis of starches isolated from unpolished red rice (URR) under a combination of germination and HMT (percent, dry basis).

**Rice starches**	**Protein**	**Lipid**	**Ash**	**Total carbohydrate**
NRS	0.136 ± 0.001^d^	0.085 ± 0.007^d^	0.826 ± 0.051^c^	98.9 ± 0.1^bc^
G‐RS‐6h	0.017 ± 0.001^e^	0.565 ± 0.045^b^	0.507 ± 0.038^de^	98.9 ± 0.1^bc^
G‐RS‐12h	0.042 ± 0.003^e^	0.137 ± 0.012^d^	0.461 ± 0.031^e^	99.4 ± 0.1^a^
G‐RS‐18h	0.018 ± 0.001^e^	0.851 ± 0.004^a^	0.329 ± 0.020^e^	98.8 ± 0.1^c^
G‐RS‐24h	0.053 ± 0.003^e^	0.123 ± 0.007^d^	0.724 ± 0.058^cd^	99.1 ± 0.1^b^
GH‐RS‐6h	0.330 ± 0.014^b^	0.146 ± 0.005^d^	1.097 ± 0.067^b^	98.4 ± 0.1^d^
GH‐RS‐12h	0.257 ± 0.002^c^	0.798 ± 0.008^a^	1.321 ± 0.083^ab^	97.6 ± 0.1^e^
GH‐RS‐18h	0.259 ± 0.026^c^	0.380 ± 0.022^c^	1.556 ± 0.110^a^	97.8 ± 0.2^e^
GH‐RS‐24h	1.815 ± 0.025^a^	0.137 ± 0.002^d^	1.278 ± 0.031^b^	96.8 ± 0.9^f^

*Note:* G‐RS‐6h, G‐RS‐12h, G‐RS‐18h, and G‐RS‐24h are rice starches isolated from the unpolished red rice after germination for 6, 12, 18, and 24 h, respectively; GH‐RS‐6h, GH‐RS‐12h, GH‐RS‐18h, and GH‐RS‐24h are rice starches isolated from the unpolished red rice after a combination of germination for 6, 12, 18, and 24 h and heat–moisture treatment, respectively. Data (means ± SD) followed by the same superscript letter in the same column show no significant difference (*p* < 0.05), *n* = 3.

Abbreviation: NRS, native rice starch.

### 3.2. Morphology of Rice Starch Granules Under Germination and HMT

Figure 1 illustrates the morphological features of red rice starch granules isolated from unpolished red rice grains under different germination durations, with and without the application of HMT. The starch granules were found to be irregular and polyhedral in shape, with granule sizes ranging from 3 to 8 *μ*m, consistent with the findings of Xu et al. [22]. The granules of native rice starch (Figure 1a) had smooth, undamaged surfaces. The morphology of rice starch granules changed gradually during germination. After 6 h of germination, the granular surface became slightly rougher and exhibited numerous pores (Figure 1b). As germination time increased, the granules displayed more pronounced surface roughening, the appearance of cracks, and larger pores, reflecting enhanced enzymatic activity (Figures 1c, 1d, and 1e), consistent with prior findings [23]. After 24 h of germination, most starch granules retained their original shape, though germination gradually affected their size, surface, and internal structure. According to Xu et al. [22], grain germination is accompanied by enzyme hydrolysis. Enzymes can penetrate the granules, generating surface pores and initiating hydrolysis from the hilum region outward, leading to the formation of holes on the surface. Extended germination times resulted in higher concentrations of hydrolytic enzymes, which were responsible for creating porosities on the granule surface [15]. These porosities were formed through enzymatic attachment and hydrolysis of the starch granules [23]. Previous research identified five patterns of enzyme activity: pinholes, sponge‐like erosion, multiple medium‐sized holes, single holes in individual granules from different loci, and surface erosion [23]. Consequently, aside from a few small pinholes observed on the surface of some starch granules, no substantial damage to germinated red rice starch was evident. This was attributed to the dissolution of highly hydrolyzed granules during extraction or the removal of eroded starch granules from the upper layer after centrifugation of starch suspensions.

Figure 1Scanning electron microscope (SEM) of starches isolated from unpolished red rice (URR) under a combination of germination and HMT. (a) Native rice starch. (b–e) Rice starch isolated from the URR under germination for 6, 12, 18, and 24 h, respectively. (f–i) Rice starch isolated from the URR under a combination of germination for 6, 12, 18, and 24 h and heat–moisture treatment, respectively.

**Figure:**
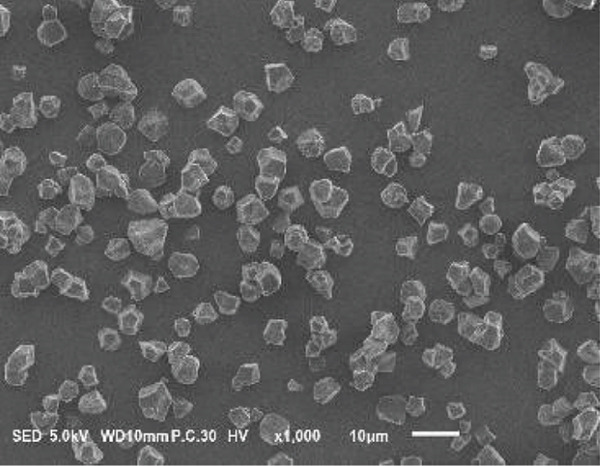
(a)

**Figure:**
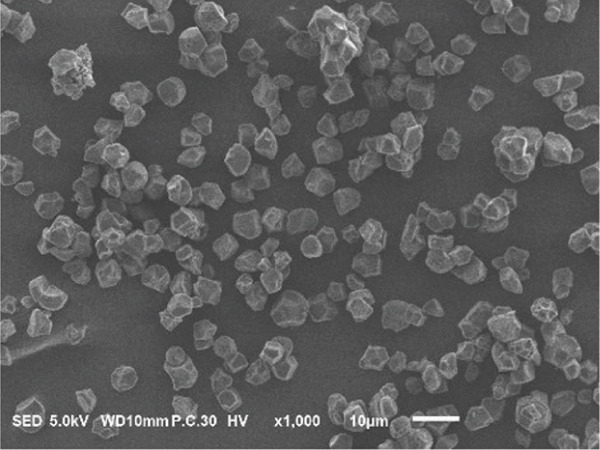
(b)

**Figure:**
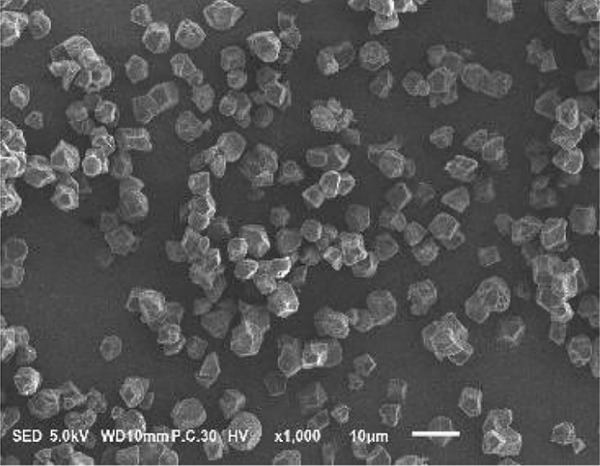
(c)

**Figure:**
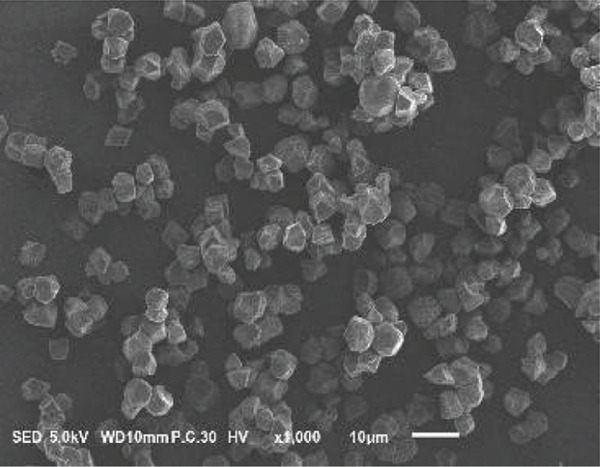
(d)

**Figure:**
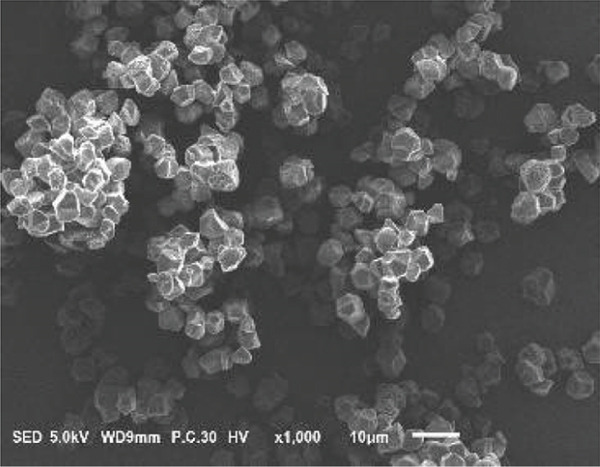
(e)

**Figure:**
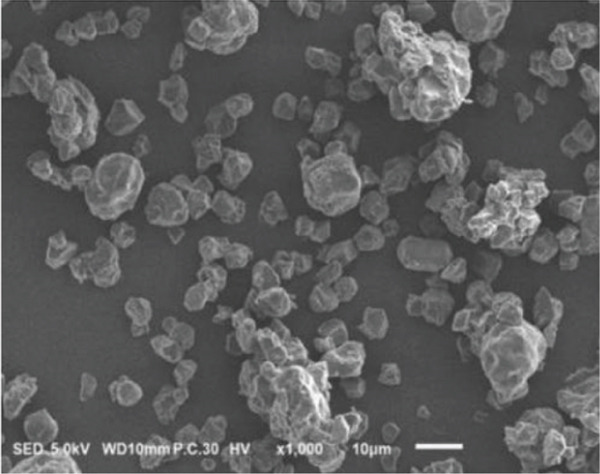
(f)

**Figure:**
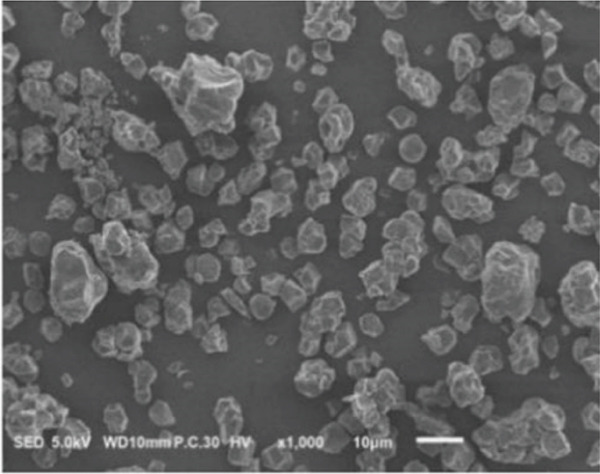
(g)

**Figure:**
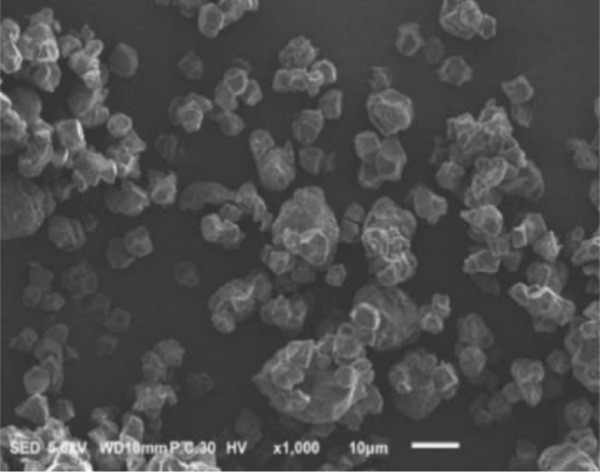
(h)

**Figure:**
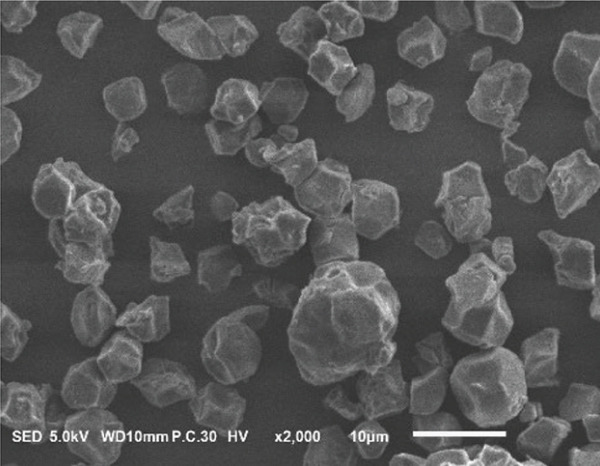
(i)

After combining germination and HMT, the starch granules of red rice no longer retained their original shape due to surface gelatinization. The pores on the granule surfaces disappeared, and the surface structure reorganized as a result of interactions between amylose and amylopectin chains. These chains reassociated after heating at high temperatures and subsequent cooling, forming crystalline complexes [24]. The size of these starch granules was also larger than that of the native starch granules, likely due to adhesion between granules. Additionally, it could be explained by the creation of starch–protein complexes after germination and HMT [21]. These results indicate that the starch granules in red rice were partially gelatinized and underwent structural changes because of the combined effects of HMT on germinated red rice.

### 3.3. X‐Ray Diffractions of Rice Starch Granules Under Germination and HMT

**Figure 2:**
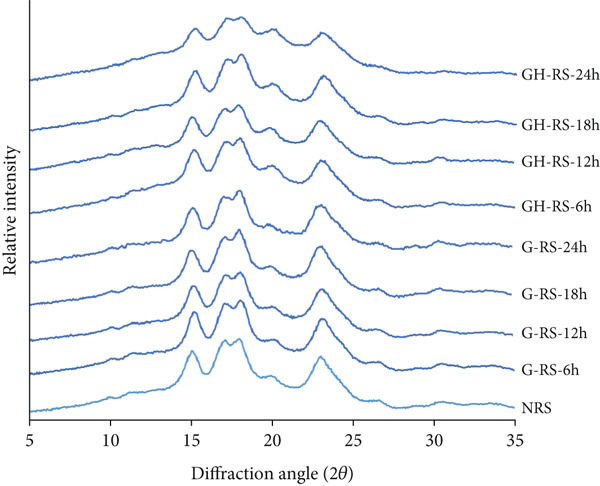
X‐ray diffraction pattern of starches isolated from unpolished red rice (URR) under a combination of germination and HMT. NRS, native rice starch; G‐RS‐6h, G‐RS‐12h, G‐RS‐18h, and G‐RS‐24h, rice starch isolated from the URR under germination for 6, 12, 18, and 24 h, respectively; GH‐RS‐6h, GH‐RS‐12h, GH‐RS‐18h, and GH‐RS‐24h, rice starch isolated from the URR under a combination of germination for 6, 12, 18, and 24 h and heat–moisture treatment, respectively.

The crystalline structure of starches obtained from untreated, germinated, and germinated‐and‐heat–moisture‐treated rice grains is shown in Figure 2. All starches derived from untreated and germinated rice grains, with or without HMT, exhibited an A‐type crystalline structure, characterized by diffraction peaks around 17°, 18°, and 23°. These findings suggest that germination and HMT had a negligible impact on the crystalline structure of starch. According to Li et al. [25], starch breakdown during germination begins in the amorphous regions of the granules, followed by the crystalline regions. Li et al. [25] further reported that the crystallinity of starch peaks after 12 h of germination but decreases as germination extends to 24 h. During the first 12 h, hydrolysis of the amorphous regions lowers the denominator in the crystallinity ratio calculation, leading to an apparent increase in crystallinity. However, prolonged germination up to 24 h likely results in gradual hydrolysis of the crystalline regions, partially disrupting the microcrystalline structure and weakening molecular chain interactions. This ultimately causes a decrease in starch crystallinity [12]. HMT induced a regular arrangement of double helices formed by amylose–amylose or amylopectin branches, leading to the creation of crystalline regions. However, because the number of double helices was much greater than that of crystallites, not all double helices of branched amylopectin contributed to the formation of these crystalline regions [14]. As a result, all starches from germinated red rice retained the same A‐type crystalline structure typical of rice starch.

### 3.4. Pasting Property of Rice Starches Under Germination and HMT

Table 2 illustrates the pasting characteristics of starches isolated from untreated, germinated, and germinated‐and‐heat–moisture‐treated rice grains. The peak viscosity, breakdown, and setback of red rice starches increased dramatically over the 24‐h germination process, whereas the final viscosity significantly decreased, and the pasting temperature remained unchanged. Similar trends have been reported by Charoenthaikij et al. [26] and Xu et al. [22]. The starch paste of 24‐h‐germinated red rice (G‐RS‐24h) exhibited the highest peak viscosity, breakdown, and setback values, while the 12‐h‐germinated red rice (G‐RS‐12h) had the lowest values for these parameters. Li et al. [27] also observed that the peak viscosities of oat and brown rice starches increased following 24 h of germination. In general, longer germination durations correspond to higher peak viscosity, breakdown, and setback, as retrogradation tendencies change over time. After HMT, the pasting temperature (or gelatinization temperature) of the treated rice starch was significantly higher than that of untreated or germinated starches, while the peak viscosity and breakdown values were notably lower. The setback, an indicator of the degree of retrogradation, also decreased. Notably, the modified rice starches had higher final viscosity compared to the corresponding germinated rice starches. HMT enhances intermolecular interactions between starch chains, strengthening the granular structure and requiring higher thermal energy to break down the treated granules. The pasting characteristics of rice starch are influenced by amylose concentration, protein and fat content, and the distribution of amylopectin branch chain lengths [28–31]. After germination, the activation of amylase produces more short‐chain starches. HMT subsequently creates more crystalline regions within the starch, leading to an increase in final viscosity compared to the corresponding germinated starches. In rice flour, proteins can hinder the breakdown of swollen granules during the pasting process. The hydrolysis of proteins disrupts the formation of a protein network, making the swollen starch granules more fragile and causing a reduction in viscosity [31].

**Table 2:** Pasting properties of starches isolated from unpolished red rice (URR) under a combination of germination and HMT.

**Rice starches**	**Pasting temperature (°C)**	**Peak viscosity (BU)**	**Trough viscosity (BU)**	**Final viscosity (BU)**	**Breakdown (BU)**	**Setback (BU)**
NRS	68.6 ± 0.7^d^	433 ± 5.7^b^	156 ± 4.2^b^	362 ± 2.8^a^	277 ± 2.8^b^	252 ± 2.8^b^
G‐RS‐6h	69.2 ± 1.0^c^	425 ± 4.2^b^	153 ± 8.5^b^	344 ± 5.7^c^	272 ± 2.8^b^	234 ± 1.4^c^
G‐RS‐12h	69.7 ± 0.7^c^	383 ± 4.2^c^	154 ± 2.8^b^	358 ± 2.8^ab^	229 ± 5.7^c^	203 ± 4.2^de^
G‐RS‐18h	69.1 ± 0.6^c^	458 ± 5.7^a^	157 ± 5.7^b^	342 ± 1.4^c^	301 ± 5.7^a^	244 ± 4.2^bc^
G‐RS‐24h	69.5 ± 0.4^c^	465 ± 2.7^a^	154 ± 5.7^b^	338 ± 4.2^d^	311 ± 4.2^a^	273 ± 4.2^a^
GH‐RS‐6h	76.3 ± 0.5^b^	311 ± 5.7^e^	169 ± 5.7^a^	361 ± 8.5^a^	142 ± 5.7^ef^	218 ± 4.2^d^
GH‐RS‐12h	78.0 ± 1.2^a^	326 ± 8.5^de^	171 ± 4.2^a^	358 ± 5.7^ab^	155 ± 2.8^e^	211 ± 4.2^d^
GH‐RS‐18h	76.9 ± 1.0^b^	344 ± 5.7^d^	170 ± 4.2^a^	350 ± 5.7^b^	174 ± 5.7^d^	194 ± 5.7^e^
GH‐RS‐24h	76.8 ± 0.1^b^	279 ± 5.7^f^	153 ± 4.2^b^	364 ± 1.4^a^	126 ± 4.2^f^	244 ± 2.8^bc^

*Note:* G‐RS‐6h, G‐RS‐12h, G‐RS‐18h, and G‐RS‐24h are rice starches isolated from the unpolished red rice under germination for 6, 12, 18, and 24 h, respectively; GH‐RS‐6h, GH‐RS‐12h, GH‐RS‐18h, and GH‐RS‐24h are rice starches isolated from the unpolished red rice under a combination of germination for 6, 12, 18, and 24 h and heat–moisture treatment, respectively. Data (means ± SD) followed by the same superscript letter in the same column show no significant difference (*p* < 0.05), *n* = 3.

Abbreviation: NRS, native rice starch.

### 3.5. Swelling Power of Rice Starches Under Germination and HMT

The swelling power of starches isolated from untreated, germinated, and combining germinated and heat–moisture‐treated rice grains is presented in Figure 3. The swelling power of native and germinated red rice starch was significantly boosted as the operating temperature rose from 50°C to 90°C. In general, germination enhanced the swelling power of germinated starches much more than that of untreated starches. The results indicate that all germinated starches exhibited significantly higher swelling power than the untreated starch when the heating temperature was in the range of 70°C–90°C. Previous studies have suggested that amylose content is inversely proportional to swelling power [32]. The dramatic rise in swelling power in germinated red rice starch occurred because germination resulted in a substantial decline in amylose content [23]. Similarly, the negative correlation between the swelling power of wheat starch and amylose content was also confirmed by Sasaki and Matsuki [33]. Thus, the increase in swelling power of starches isolated from germinated rice grains can be explained by the reduction in amylose content during germination [23]. After HMT, the swelling power of starch isolated from the treated rice grains was significantly lower than that of the untreated starch. The decrease in swelling power of the treated starches was due to the internal starch chain rearrangement in the crystalline region, resulting in the formation of more ordered double‐helical amylopectin side‐chain clusters. In addition, the formation of lipid–amylose complex chains also suppressed the swelling power of starches [34].

Figure 3Swelling power (grams per gram) of starches isolated from unpolished red rice (URR) under (a) germination and (b) a combination of germination and HMT. NRS, native rice starch; G‐RS‐6h, G‐RS‐12h, G‐RS‐18h, and G‐RS‐24h, rice starch isolated from the URR under germination for 6, 12, 18, and 24 h, respectively; GH‐RS‐6h, GH‐RS‐12h, GH‐RS‐18h, and GH‐RS‐24h, rice starch isolated from the URR under a combination of germination for 6, 12, 18, and 24 h and heat–moisture treatment, respectively.

**Figure:**
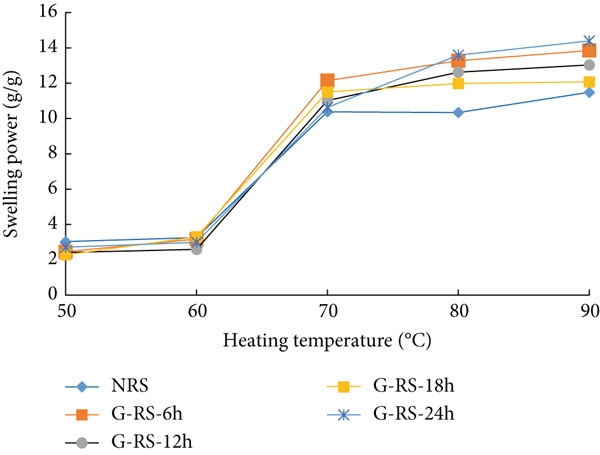
(a)

**Figure:**
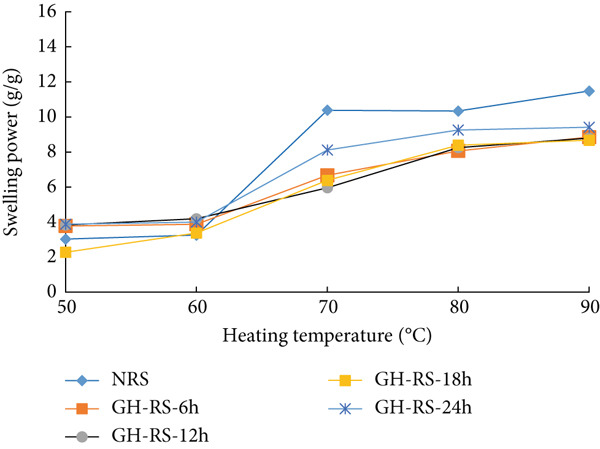
(b)

### 3.6. Solubility of Rice Starches Under Germination and HMT

The solubility of starches isolated from untreated, germinated, and combined germinated and heat–moisture‐treated rice grains is presented in Table 3. Similar to the swelling power, an increase in the solubility of native, germinated, and HMT rice starches was clearly observed as the heating temperatures increased from 70°C to 90°C, reaching a maximum value at 80°C. The solubility of germinated rice starches significantly decreased with longer germination periods, particularly when the heating temperature exceeded 80°C. A similar trend of reduced solubility was also observed for the combined germinated and heat–moisture‐treated rice starches with extended germination times, although the solubility of the combined germinated and heat–moisture‐treated rice starches was remarkably higher than that of the germinated rice starches at the same germination duration and heating temperature. The difference in starch solubility might be attributed to structural variations, as deviations in solubility are caused by differences in chain length distributions. The decrease in solubility of rice starches with longer germination time may be due to the consumption of short‐chain amylose by the sprouts [12]. In contrast, the increase in starch solubility after HMT might be attributed to the process facilitating the connection of amylose molecules in the bulk amorphous regions with branching amylopectin segments in the crystalline regions [35], which promotes the dispersion of starch molecules in water [36]. Additionally, the structure on the surface of starch granules might be weakened, thereby increasing solubility. Furthermore, amylose–lipid complexation and the structure of amylopectin also contributed to the changes in solubility of rice starches after germination and HMT [37].

**Table 3:** Solubility (percent) of starches isolated from unpolished red rice (URR) under a combination of germination and HMT.

**Rice starches**	**Heating temperature (°C)**				
	**50**	**60**	**70**	**80**	**90**
NRS	1.23 ± 0.01^e^	3.58 ± 0.21^c^	9.51 ± 0.35^c^	15.3 ± 0.2^f^	14.4 ± 0.4^d^
G‐RS‐6h	1.63 ± 0.28^d^	4.88 ± 0.13^a^	10.1 ± 0.2^b^	14.5 ± 0.2^f^	13.3 ± 0.1^e^
G‐RS‐12h	1.20 ± 0.07^e^	4.56 ± 0.49^a^	6.39 ± 0.51^f^	13.2 ± 0.3^g^	11.1 ± 0.1^f^
G‐RS‐18h	1.20 ± 0.01^e^	4.54 ± 0.35^a^	8.69 ± 0.08^e^	13.9 ± 0.1^g^	13.9 ± 0.1^d^
G‐RS‐24h	1.22 ± 0.02^e^	3.64 ± 0.06^c^	8.81 ± 0.16^e^	11.6 ± 0.2^h^	10.8 ± 0.2^d^
GH‐RS‐6h	1.88 ± 0.04^c^	3.03 ± 0.02^d^	11.8 ± 0.3^a^	20.3 ± 0.3^a^	18.6 ± 0.5^a^
GH‐RS‐12h	1.82 ± 0.04^c^	2.93 ± 0.14^d^	10.4 ± 0.1^b^	19.5 ± 0.2^b^	17.4 ± 0.7^b^
GH‐RS‐18h	2.44 ± 0.04^b^	2.61 ± 0.15^e^	9.88 ± 0.17^c^	17.5 ± 0.5^c^	16.0 ± 0.1^c^
GH‐RS‐24h	3.25 ± 0.29^a^	3.94 ± 0.36^b^	9.00 ± 0.36^d^	16.3 ± 0.4^e^	15.7 ± 0.6^c^

*Note:* G‐RS‐6h, G‐RS‐12h, G‐RS‐18h, and G‐RS‐24h are rice starches isolated from the unpolished red rice under germination for 6, 12, 18, and 24 h, respectively; GH‐RS‐6h, GH‐RS‐12h, GH‐RS‐18h, and GH‐RS‐24h are rice starches isolated from the unpolished red rice under a combination of germination for 6, 12, 18, and 24 h and heat–moisture treatment, respectively. Data (means ± SD) followed by the same superscript letter in the same column show no significant difference (*p* < 0.05), *n* = 3.

Abbreviation: NRS, native rice starch.

### 3.7. In Vitro Digestibility of Rice Starches Under Germination and HMT

**Figure 4:**
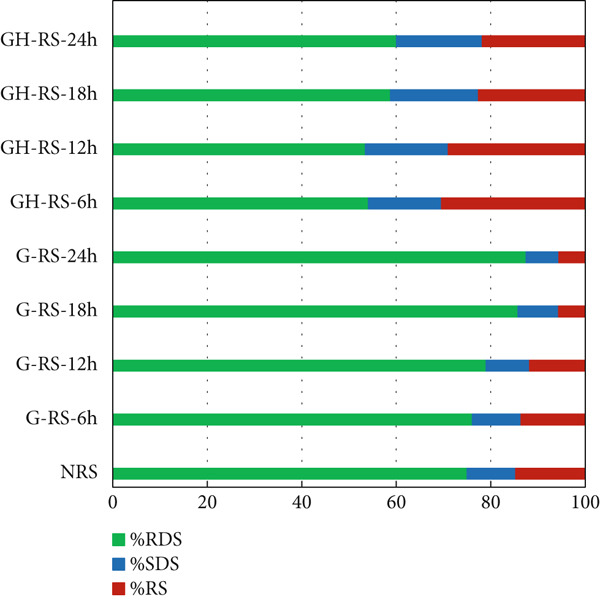
Starch fractions (RDS, SDS, and RS, percent, dry basis) of starches isolated from unpolished red rice (URR) under a combination of germination and HMT. NRS, native rice starch; G‐RS‐6h, G‐RS‐12h, G‐RS‐18h, and G‐RS‐24h, rice starch isolated from the URR under germination for 6, 12, 18, and 24 h, respectively; GH‐RS‐6h, GH‐RS‐12h, GH‐RS‐18h, and GH‐RS‐24h, rice starch isolated from the URR under a combination of germination for 6, 12, 18, and 24 h and heat–moisture treatment, respectively.

Figure 4 presents the concentrations of RDS, SDS, and RS characterized from starches isolated from untreated, germinated, and combined germinated and heat–moisture‐treated rice grains. The starches isolated from germinated red rice exhibited higher amounts of RDS (76.1%–87.5%) but lower concentrations of SDS (6.93%–10.31%) and RS (5.57%–13.59%) compared to those of native rice starch. The increase in RDS levels and the reduction in SDS and RS levels were positively correlated with the germination duration, which ranged from 6 to 24 h. These findings align with previous research, which confirmed that germination improves rice digestibility [22]. The observed improvements in digestibility were primarily due to the activity of amylolytic enzymes [15]. During germination, these enzymes effectively degraded starch chains into readily digestible products, resulting in increased RDS concentrations and reduced RS amounts. Xu et al. [22] also confirmed that the germination process could reduce the amylose content in starch, thereby enhancing its digestibility.

After combining germination and HMT, the proportions of RDS in the treated rice starches significantly reduced (53.48%–60.03%), whereas their SDS and RS contents significantly improved (15.51%–18.60% and 21.79%–30.39%, respectively) compared to those of germinated rice grains. These outcomes align with the findings of Hung et al. [14], which reported a reduction in RDS content and an improvement in RS levels in heat–moisture‐treated red rice. The variations in RDS and RS contents of red rice starches can be attributed to structural changes in starch granules during HMT [15]. According to Chung et al. [34], increased interactions between starch chains, amylose retrogradation upon cooling, and the development of physical cross‐links in amorphous regions enhance the perfection of existing starch crystallites, leading to an increase in RS and SDS concentrations during HMT. Consequently, HMT of germinated rice grains induces structural changes in starch granules and molecules, making them less susceptible to digestive enzymes.

## 4. Conclusion

The sequential application of germination followed by HMT on unpolished red rice had a significant influence on both the digestibility and physicochemical properties of its starches. These treatments did not significantly alter the crystalline structure of red rice starch, which remained in the A‐type category. Although the starch granules isolated from native, germinated, and combined germinated and heat–moisture‐treated red rice were all irregular and polyhedral in shape, their porosity and the number of surface holes were dependent on the germination duration. Pasting properties, including pasting temperature, maximum viscosity, and breakdown, were also affected by both germination and HMT. Germination had the potential to enhance the swelling power and reduce solubility, whereas HMT had the opposite effect on these characteristics. Moreover, when HMT was applied after germination, the RS content of red rice starches increased, while a decline was observed in the amount of RDS.

## Ethics Statement

This article does not contain any studies with human or animal subjects.

## Conflicts of Interest

The authors declare no conflicts of interest.

## Author Contributions

Le Thi Thu Suong and Tran Ngoc My Linh: methodology, investigation, formal analysis, and writing—original draft preparation. Nguyen Thi Lan Phi and Nguyen Ngoc Thanh Tien: conceptualization, resources, methodology, and writing—original draft preparation. Mai Nguyen Tram Anh and Chau Minh Thuan: methodology, validation, and formal analysis. Pham Van Hung: conceptualization, investigation, methodology, supervision, validation, and writing—reviewing and editing. Tran Ngoc My Linh and Le Thi Thu Suong share equal work.

## Funding

This study was funded by Viet Nam National University Ho Chi Minh City (10.13039/501100010712) (A2024‐28‐02).

## Data Availability

The data that support the findings of this study are available from the corresponding author upon reasonable request.
